# The Leading Concerns of American Women with Nausea and Vomiting of Pregnancy Calling Motherisk NVP Helpline

**DOI:** 10.1155/2013/752980

**Published:** 2013-04-15

**Authors:** Svetlana Madjunkova, Caroline Maltepe, Gideon Koren

**Affiliations:** The Motherisk Program, Division of Clinical Pharmacology/Toxicology, The Hospital for Sick Children, The University of Toronto, 555 University Avenue, Toronto, ON, Canada M5G 1X8

## Abstract

*Background*. Nausea and vomiting of pregnancy (NVP) is the most common medical condition of pregnancy, affecting up to 85% of expecting mothers. In the USA, there is no FDA-approved medication for the treatment of NVP. *Objective*. To identify the primary concerns of American women leading them to contact the Motherisk NVP Helpline and to characterize the severity of their symptoms and therapy offered in order to develop improved and customized counseling for them. *Methods*. We reviewed the intake forms of the American women who called the NVP Helpline from 2008 to 2012. We extracted their state of residence, demographic data, severity of NVP symptoms, and other available clinical characteristics. *Results*. A total of 195 forms were reviewed. Of these, 86% called for information on management of NVP with/without questions about fetal drug safety, while 14% called solely about drug safety during pregnancy/breastfeeding. The majority of women were Caucasian, in their thirties, educated, employed, married and in their second pregnancy. Of them 95% were suffering from moderate-to-severe condition with 13% having hyperemesis gravidarum. *Conclusion*. American women need more information on the management of NVP and on a variety of its aspects in addition to the safety and effectiveness of antiemetic medications. Their leading concern was the use of doxylamine and vitamin B6 combination for NVP treatment followed by the use of ondansetron.

## 1. Introduction

Since the removal of Bendectin (doxylamine-pyridoxine delayed release combination) from the American market in 1983, pregnant women in the USA remained without an FDA-approved medication to manage the most common medical condition of pregnancy, affecting up to 85% of expecting mothers [1]. This move resulted in a 3-fold increase in hospitalization rate for severe NVP in the USA in the period from 1983 to 1988, a trend not seen in Canada, where this antiemetic combination continued to be prescribed [2].

In 1995, we established the first and presently the only NVP Helpline, counseling expecting and planning mothers on the management of NVP. The counseling is evidence based and updated continuously with the available data from research and systematic review of clinical and experimental publications. The NVP Helpline number 1-800-436-8477 is toll free for Canada and the USA [3, 4]. 

Women often report to us that their health care providers tend to trivialize and belittle the seriousness of NVP, claiming it is a natural part of pregnancy, causing large numbers of them to be suboptimally managed. Women calling Motherisk report that American physicians often try to avoid all medication in pregnancy, particularly during the first trimester. Moreover, physicians often are reported to say that they are not worried about the women medically, unless the woman is losing weight and has ketones in the urine, which are some of the symptoms related to the most severe end of NVP called hyperemesis gravidarum (HG).

The objective of the present study was to identify the primary concerns of American women, leading them to contact our NVP Helpline, to characterize the severity of their symptoms and therapy offered to them and to develop improved and customized counseling for them. The data content of the counseling are followed by the evidence-based information developed by the Motherisk program relevant to the queries by American women.

## 2. Methods

We reviewed the intake forms of American women who called the Motherisk NVP Helpline from January 2008 to July 2012. After enquiring about characteristics of the caller, we shared with her evidence-based information regarding management options for NVP.

From the computerized intake forms, we extracted their state of residence in the US, their ages, gravidity, parity, severity of NVP symptoms as measured by the validated PUQE-24 (pregnancy-unique quantification of emesis scores), and other available clinical characteristics. The focus of interest surrounded the main cause for their calls to our service.

Descriptive statistics were used as relevant. 

## 3. Results

A total of 195 forms were reviewed. Of these, 167 (86%) called for information how to manage NVP symptoms with or without questions about fetal drug safety info, while 28 of 195 (14%) called only about drug safety during pregnancy or breastfeeding. Their geographic distribution within the USA is given in Table 1, showing callers contacting the service from all parts of the USA. Their demographic characteristics are given in Table 2. 

**Figure 1:**
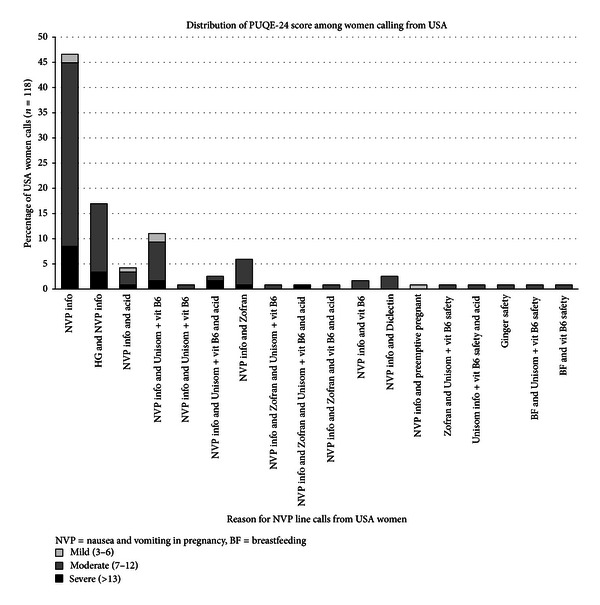
Distribution of PUQE-24 score among women calling from USA.

Table 3 presents the severity of NVP symptoms among the callers, using the validated PUQE-24 scores [5], showing most callers suffering from moderate-to-severe conditions, probably leading them to seek advice to try to improve their condition. Distribution of PUQE-24 scores among women calling from USA is given in Figure 1.

The following is the breakdown of the 167 women who called for NVP management information only/and drug safety info.

About 30% of all calls (58/195) were specifically for NVP management counseling. Of all women, 13% (25/195) had HG at time of the call and needed NVP management counseling. 

35% (68/195) of the women called for NVP management counseling as well as for safety information on drugs used for controlling nausea or vomiting. 

Distribution of gestational ages of the callers at time of contact is presented in Table 4.

Almost 6% (11/195) of all calls were from women who experienced acid reflux/dyspepsia symptoms. These women received from us NVP management counseling related to the management of hyperacidity.

4/195 of the callers (2%) were interested in our preemptive protocol, starting antiemetics before symptoms erupted, after experiencing severe NVP in a previous pregnancy. Two of these women were planning pregnancy and had a history of HG in their previous pregnancy/ies, and two were already pregnant but not yet with symptoms of NVP.

The breakdown of the 28 (14%) women who called only regarding drug safety in pregnancy or breastfeeding is presented in Table 5.

The following section describes the advice given to the American callers. 

## 4. Basic NVP Counseling

Up to 85% of women will have nausea and/or vomiting in pregnancy between 12 and 16 weeks, and 20% of women will experience symptoms up to 5 months of pregnancy or time of delivery. The range of severity of NVP symptoms can vary from mild to severe. The most severe form of NVP is hyperemesis gravidarum, that affects up to 2% of pregnant women. Nausea and/or vomiting could start as early as 4 weeks up to the 9th week of pregnancy. Symptoms can peak anywhere from 7 to 12 weeks of pregnancy.

The following recommendations are based on research studies and clinical experience. Any addition or changes to the medical treatment should always be discussed with and recommended by a healthcare provider. The dietary recommendations should only be followed if there are no dietary restrictions due to allergies or medical conditions.

### 4.1. Dietary and Lifestyle Suggestions

- Divide portions and eat smaller amounts every 1 to 2 hours. Do not skip meals. Add any source of protein to each snack or meal, which will help regulate blood sugar levels and may lessen the morning sickness.
- Keep the consumption of solids and liquids separate. Drink fluids 20 to 30 minutes before and after snacks/meals.
- Try to increase fluid intake up to 8 cups/day (2 liters/day).
- Drink colder fluids, including ice chips, popsicles, or slushies. They are easier to tolerate and will maintain your hydration. It may also help in decreasing the metallic taste in mouth.
- To help increase fluid intake and to prevent getting dehydrated, you may also add electrolytes (e.g., sports drinks, vitamin water, or coconut water).
- If unable to keep food down or for extra nutrients, consider adding liquid supplements (protein or nutritional), puddings or bars to your diet. However, do not replace any meals with them.
- Discuss with your health care provider the use of prenatal vitamins with lower iron content in the first trimester as the iron in the supplement may cause additional nausea, vomiting, upset stomach or constipation. If you have low iron levels, you may try splitting the vitamin, taking half in the morning and half in the evening. If your iron level is normal, try switching to a children's chewable vitamin and add folic acid. Iron, for both mother and baby, is needed after 12 weeks.
- Symptoms of heartburn, reflux or indigestion may aggravate or increase morning sickness. Talk to your health care provider for possible treatment options such as calcium carbonate, H_2_ blockers, or proton pump inhibitors.
- Symptoms of gas, bloating, and/or lactose intolerance are common in pregnancy; try switching to lactose-free products. Talk to your health care provider for possible treatment options such as antiflatulent agents.
- The incidence of constipation in pregnancy is common and could be aggravated from lack of fluids, prenatal vitamins and/or lack of fiber in your diet. Try increasing dietary fiber intake along with fluid intake. Talk to your health care provider for possible treatment options such as stool softeners.
- In case having excess of saliva, it may be helpful to spit it out and to do frequent mouth washing. By swallowing it, nausea and/or vomiting may increase.
- Try not to brush your teeth right after eating.
- If you have a bitter or metallic taste in your mouth, try hard candies, chew gums, and suck on lemons or limes.
- If you have a heightened sense of smell, try to ventilate; eat meals lukewarm/cold or sniff things like lemons, limes, oranges, candles, and cinnamon sticks and so on.

### 4.2. Non-Medical Suggestions

Non-medical treatments have been commonly used to treat morning sickness. Some have been studied in pregnancy such as ginger, vitamin B6, acupressure, and acupuncture. It is important to discuss with your health care provider any of the non-medical treatments you may consider as some may interfere with other medications or may be harmful during pregnancy.

You may add at any time the following:

- Sea bands or acupressure bands (used for motion sickness).
- Vitamin B6, 50 mg tablet, 3-4x a day (up to 200 mg a day) [6].
- Ginger root powder capsules, 250 mg, 4 times a day or 500 mg 2 times a day (dried ginger root powder equivalent). Ginger may cause gastric irritation and should not be taken on an empty stomach as it may aggravate your symptoms.

These non-medical approaches should be tried for 4 days, either individually or combined. If helpful, continue use as long as you need it for, if not discontinue use.

### 4.3. Motherisk Statements (“Safety”) on Medications Used in Pregnancy [7]

For every pregnancy, there is a baseline risk of 1–3% for having a baby with birth defect by chance alone. This applies for all pregnancies, not counting the medical conditions or maternal exposures. 

#### 4.3.1. Diclectin

Diclectin is the drug of choice for nausea and vomiting in pregnancy in Canada which is composed of 2 ingredients: vitamin B6 (pyridoxine, 10 mg) and doxylamine succinate (antihistamine, 10 mg). The delayed release formulation of Diclectin requires the prescribed dose to be taken daily for optimal effect. It's been on the Canadian market for over 30 years, and it is the only medication labeled for pregnancy by the government of Canada for over 20 years. It is the most studied drug formulation in pregnancy to date. Studies have shown that Diclectin will not increase your baseline risk for major malformations (1–3%) even if taken in the first trimester and has not been associated with any adverse pregnancy outcomes [7]. 

It may cause sedation, tiredness, or dizziness. 

#### 4.3.2. Dimenhydrinate, Meclizine, and Doxylamine

Dimenhydrinate, meclizine and doxylamine are antihistamines from the group of H_1_ blockers. This class of antihistamines has been studied by case control cohorts, prospective studies, and meta-analysis (on over 24 studies) on thousands of pregnancy exposures which did not show any increase risk of major malformations above the baseline risk (1–3%) [7].

However, this group of medications may also cause sedation, tiredness, or dizziness. 

#### 4.3.3. Promethazine

Phenothiazines, such as prochlorperazine, promethazine, and chlorpromazine, are commonly used antiemetics and antipsychotics. With regards to NVP/HG, numerous studies have not shown an increased risk for major malformations. When used continuously into the third trimester, neonatal withdrawals, including extrapyramidal effects, have been reported in newborns [7].

#### 4.3.4. Ondansetron

Ondansetron is a selective serotonin 5-HT3 receptor antagonist known for its use in treating chemotherapy-related nausea and vomiting. Despite its cost and limited safety profile, it is commonly used. 

A recent large case control study detected a 2-fold increased risk of cleft palate associated with ondansetron taken for NVP in the first trimester of pregnancy (odds ratio from 2.37, 95% CI 1.28, to 4.76). However, the study has limitations and needs further clarification [8].

FDA issued a warning in September 2011 [9] about possible serious QT prolongation and torsade de pointes among people receiving ondansetron, especially ones having underlying heart diseases. It is advised to do strict followup of the patients receiving ondansetron to rule out long QT syndromes, electrolyte imbalance, and congestive heart failure or receiving concomitant medications that prolong the QT interval. ECG monitoring is recommended in these patients. In the context of NVP, quite few women with severe NVP/HG might have electrolyte imbalances (hypokalemia or hypomagnesaemia). 

Constipation is a common side-effect; however, headaches, fatigue, and drowsiness have also been reported.

### 4.4. Motherisk Statements on Medications Used in Pregnancy While Breastfeeding

#### 4.4.1. Doxylamine

Doxylamine is an H_1_ antihistamine with sedative properties, and levels in breast milk are not known. All H_1_ antihistamines generally transfer in minimal amounts and have not been associated with any concerns. There are no reports of adverse reactions in breastfed infants, but they should be observed for sedation and paradoxical CNS stimulation with caution particularly in infants with apnea or other respiratory syndromes [10].

#### 4.4.2. Vitamin B6 (Pyridoxine)

Pyridoxine is readily transferred into breast milk, and the maternal plasma concentrations reflect the breast milk concentrations. The maximal daily dose should not exceed 100 mg [11].

If needed please have a look at our NVP treatment algorithm on our Motherisk website (http://www.motherisk.org/).

## 5. Discussion

With no FDA-approved medication for NVP, and in the wake of very hostile medico legal atmosphere, many pregnant women report that their physicians are hesitating to treat their symptoms of morning sickness. Our analysis reveals that women need information on a variety of aspects of the condition, with safety and effectiveness of medications being the most common. The leading cause is the use of doxylamine and vitamin B6 in an attempt to emulate the delayed release formulation of these drugs, which have been removed from the American market in 1983. Of interest, large numbers of American women use ondansetron, although this medication was never approved and labeled for pregnancy, and there are concerns about its ability to induce cardiac dysrhythmias [12]. 

It is evident that American women need much more access to medical information pertaining to the symptoms of NVP, the most common medical condition in pregnancy.

It is hoped that including such information herein will help achieving this task.

## Figures and Tables

**Table 1:** Geographic distribution of the callers from USA.

State	Number of calls	%
New York	26	13.3%
California	20	10.3%
Florida	14	7.2%
Texas	12	6.2%
Pennsylvania	10	5.1%
Georgia	9	4.6%
Indiana	8	4.1%
New Jersey	8	4.1%
Virginia	7	3.6%
Massachusetts	6	3.1%
Michigan	6	3.1%
Ohio	6	3.1%
Wisconsin	6	3.1%
Arizona	5	2.6%
Colorado	5	2.6%
Washington	5	2.6%
Illinois	4	2.1%
North Carolina	4	2.1%
Utah	4	2.1%
Maryland	3	1.5%
Minnesota	3	1.5%
Missouri	3	1.5%
New Mexico	2	1.0%
Oregon	2	1.0%
South Carolina	2	1.0%
Tennessee	2	1.0%
Alabama	1	0.5%
Connecticut	1	0.5%
Hawaii	1	0.5%
Iowa	1	0.5%
Kansas	1	0.5%
Louisiana	1	0.5%
Maine	1	0.5%
Montana	1	0.5%
Nevada	1	0.5%
Rhode Island	1	0.5%
South Dakota	1	0.5%
Unspecified	2	1.0%

Total	195	100.0%

**Table 2:** Demographic characteristics of the 195 USA callers.

		NVP/NVP and safety (*n* = 167)		Safety of medications (*n* = 28)	
			%		%
Gestational age (gestational weeks)	Average	9.08		9.64	
	St. Dev.	3.67		3.94	
	Min	4.00		5.00	
	Max	27.50		20.00	

Maternal age (years)	Average	31.69		32.30	
	St. Dev.	5.98		5.88	
	Min	18.20		18.00	
	Max	45.00		40.00	

Maternal weight (lb)	Average	140.15		142.38	
	St. Dev.	29.01		30.01	
	Min	94.00		104.00	
	Max	305.00		226.00	

Ethnicity	Black	10	6.0%	—	
	Caucasian	38	22.8%	—	
	Hispanic	8	4.8%	—	
	Asian	1	0.6%	—	

Education	High school	13	7.8%	—	
	College	5	3.0%	—	
	University	12	7.2%	—	
	Postgraduate	14	8.4%	—	

Marital status	Single	5	3.0%	—	
	Living with partner	6	3.6%	—	
	Married	66	39.5%	—	

Employment status	Unemployed	3	1.8%	—	
	Homemaker	25	15.0%	—	
	Employed	39	23.4%	—	

Gravidity (number of pregnancies)	Gravidity 1	42	25.1%	8	28.6%
	Gravidity 2	52	31.1%	7	25.0%
	Gravidity 3	28	16.8%	8	28.6%
	Gravidity 4	23	13.8%	2	7.1%
	Gravidity ≥ 5	21	12.6%	2	7.1%

Parity (number)	Parity 0	48	28.7%	9	32.1%
	Parity 1	72	43.1%	11	39.3%
	Parity 2	29	17.4%	4	14.3%
	Parity 3	8	4.8%	2	7.1%
	Parity 4	4	2.4%	1	3.6%
	Parity ≥ 5	5	3.0%	0	0.0%

**Table 3:** Overall PUQE-24 and well-being scores from USA callers.

PUQE-24	Number of calls	%	Well-being (0–10)
Mild (3–6)	6	5.08%	4.3
Moderate (7–12)	91	77.12%	4.9
Severe (>13)	21	17.80%	4

Total calls	118		102

**Table 4:** Breakdown of the 68 women calling for NVP management info/and safety information on drugs used for controlling nausea or vomiting.

Reason for initial call	*n*	%	GA (GW ± St. Dev.)	GA range (GW)
NVP info and Unisom + vit B6	26	13.33	11.98 ± 2.89	4–21
NVP info and Zofran	16	8.21	11.77 ± 6.25	2–22
NVP info and Unisom + vit B6, and acid	9	4.62	7.61 ± 1.02	7–10
NVP info and Unisom	4	2.05	8.17 ± 0.58	7.5–8.5
NVP info and vit B6	3	1.54	8.5 ± 1.41	7.5–9.5
NVP info and Diclectin	3	1.54	7.33 ± 0.76	6.5–8
NVP info and nonmedical advice	2	1.03	7	
NVP info and Zofran and Unisom + vit B6 and acid	2	1.03	6	
NVP info and Zofran and Unisom + vit B6	1	0.51	11	
NVP info and Zofran and vit B6, and acid	1	0.51	—	
NVP info, dimenhydrinate, meclizine, and acid	1	0.51	8	

Total	68	35%		

GW: Gestational weeks.

**Table 5:** Breakdown of the 28 women calling for safety information on drugs used for controlling nausea or vomiting.

Reason for initial call	*n*	%	GA (GW ± St. Dev.)	GA range (GW)
Unisom + vit B6 info	12	6.15%	7.33 ± 1.92	5–11
Zofran and Unisom + vit B6	5	2.50%	10.75 ± 5.12	6–18
Unisom info	3	1.54%	7.67 ± 3.21	4–10
Promethazine	2	1.03%	10	10
Unisom info + vit B6 and acid	1	0.51%	6	
vit B6	1	0.51%	10	
Unisom and insomnia	1	0.51%	20	
Breastfeeding and Unisom + vit B6	1	0.51%	8	
Breastfeeding and vit B6	1	0.51%	7	
Ginger safety	1	0.51%	8.5	

Total	28	14%		

GW: Gestational weeks.

## Abbreviations

HG: Hyperemesis gravidarum
NVP: Nausea and vomiting of pregnancy
PUQE: Pregnancy-unique quantification of emesis.
